# Extracting Teeth

**Published:** 1862-08

**Authors:** W. H. Atkinson


### THE DENTAL REGISTER OF THE WEST.

VOL. XVI.] AUGUST, 1862. [No. 8.

### Original Essays and Communications.

### EXTRACTING TEETH.

BY DR. W. H. ATKINSON.

Read before the N. Y. Society of Dental Surgeons, May 14, 1862.

1. WHEN should teeth be extracted?
2. How should they be extracted?
3. For what should they be extracted?
4. When and how are they usually extracted?

I will endeavor to answer the last query first, by saying that they are almost universally extracted to get rid of annoyance to patient and operator, irrespective of the true consideration of their soundness or usefulness in the mouth, especially of children and youths.

An epidemic of destructive energy has supervened upon the ancient admonition to never remove a tooth until it could be readily removed with a pair of leaden forceps; a hint that we would do well to heed, even in these days of vaunted advancement and science.

Doubtless there are organs without which we may *seem to live*, while there are those which are indispensable to even apparent continuance of vital function.

But all the organs are necessary to completeness, and therefore we are less a man or a woman in the ratio of the subtractions which have mutilated us.

I know it has been customary to clip hair and nails, extract teeth and shed torrents of blood, all for the supposed good and comfort of those who have been submitted to these fractionalizing processes.

Custom is not always founded upon the soundest principles of philosophy more than all the operations performed upon human subjects are freed from the stimulus of pecuniary gain.

The advocates of close clipping and shaving stoutly aver that it is for the well-being of the victim; so the phlebotomist is very certain that there is nothing so good for his dupe as to be relieved of the excess of vital pabulum; and the expert mutilators of the coming men and women who are to bless or curse, in the ratio of completeness or incompleteness, the future of society, are confident that they can help madam nature to get rid of the temporary teeth much more promptly than her tedious and lingering processes of absorption and growth of the roots of the primary and crowns of the permanent set can possibly accomplish the desired result.

The advocates of the "*radical* and effectual cure of toothache" by indiscriminate extraction really esteem themselves to be great philosophers and philanthropists, whose praise is upon the tongues of so many who are but too grateful for being released from the torture of the heroic instruments of "radical cure," to take time to think that their thankfulness springs from the cessation of *pain*, not for the mutilations they will have abundant cause to regret in the aftertime.

Teeth should be extracted when nothing else will restore tranquillity to the disturbed system, and then only. They then should be extracted dexterously and painlessly as possible by the anæsthesia of skill and compatibility, not by unkind harshness and brute force.

I have never known true neuralgic toothache to be cured even by promptly extracting the tooth or teeth supposed to ache. Hence, we should be very certain that the pain is dependent upon local vice of the tooth, and the system so shattered as to have permitted a local and primarily trivial matter of symptomatic character to assume the grave character of a secondarily idiopathic disease, from which radiated the aberrant actions of which the patient suffers.

The whole subject of extracting teeth may be summed up in a few direct questions and answers.

1. Should *teeth* ever be extracted ?

*Ans.* Scarcely.

2. Should parts of teeth be extracted?

*Ans.* Sometimes, 1st, to remove causes of local bad conditions which may lead to malign influence ; 2d, to secure a sound base for artificial substitutes.

3. How may we avoid the necessity to extract?

*Ans.* By careful study of the evolution and proper arrangement of, first, the deciduous and then the permanent sets. If children were under the care of competent skill, not one in a hundred would ever need artificial dentures : Therefore, the sooner parents and guardians of the dear little ones put them under the charge of the proper person, the sooner will the year of jubilee be inaugurated, in which it will be a coveted privilege to visit the urbane dentist for his invaluable advice and little gentle manipulations which will then be necessary, instead of the dreaded penance of broken law that it too often now is to both adult and child.

These things ought not so to be, and if we who have the high honor of bending the twigs of humanity will only set about it in right good human earnest, we shall soon see the fruits of our labor and be satisfied.

